# Supplementary material for: Network Science Based Quantification of Resilience Demonstrated on the Indian Railways Network
Source: PLoS One. 2015 Nov 4;10(11):e0141890. doi: 10.1371/journal.pone.0141890 (PMC4633230; doi:10.1371/journal.pone.0141890)
Supplement: S1 Table — (DOCX) [file pone.0141890.s001.docx]

**S1 Table: List of the stations removed for each of the three hazard**s

| **Tsunami** | **Power Failure** | **Cyber-Physical** |
| --- | --- | --- |
| Bhubaneswar | Ajmer | Ahmedabad |
| Chennai Central | Ambala Cant | Bangalore City |
| Chennai Egmore | Amritsar | Chennai Central |
| Cuttack | Anand Vihar | Chennai Egmore |
| Ernakulam | Asansol | Delhi |
| Gudivada | Bareilly | H Nizamuddin |
| Guntur | Bareilly City | Howrah Jn |
| Kanyakumari | Bikaner | Hyderabad Decan |
| Kochuveli | Danapur | Jaipur |
| Kollam Jn | Darbhanga | Kacheguda |
| Machilipatnam | Delhi | Kolkata |
| Madurai | Delhi S Rohilla | Lokmanyatilak |
| Mayiladuthurai | Dibrugarh | Mumbai |
| Nagercoil | Dibrugarh Town | New Delhi |
| Narasapur | Firozpur Cant | Pune |
| Puducherry | Ghaziabad | Sealdah |
| Puri | Gorakhpur | Secunderabad |
| Rameswaram | Guwahati | Shalimar |
| Sengottai | H Nizamuddin | Yesvantpur |
| Tiruchendur | Howrah |  |
| Tiruchirapalli | Jaipur |  |
| Tirunelveli | Jammu Tawi |  |
| Tirupati | Kamakhya |  |
| Trivandrum | Kanpur Anwrganj |  |
| Tuticorin | Kanpur Central |  |
| Vijayawada | Kolkata |  |
| Villupuram | Lal Kuan |  |
| Visakhapatnam | Lucknow |  |
|  | Lucknow |  |
|  | Muzaffarpur |  |
|  | New Delhi |  |
|  | New Tinsukia |  |
|  | Palwal |  |
|  | Patna |  |
|  | Pratapnagar |  |
|  | Puri |  |
|  | Sealdah |  |
|  | Tinsukia |  |
|  | Varanasi |  |
